# DISC1 complexes with TRAK1 and Miro1 to modulate anterograde axonal mitochondrial trafficking

**DOI:** 10.1093/hmg/ddt485

**Published:** 2013-10-02

**Authors:** Fumiaki Ogawa, Elise L.V. Malavasi, Darragh K. Crummie, Jennifer E. Eykelenboom, Dinesh C. Soares, Shaun Mackie, David J. Porteous, J. Kirsty Millar

**Affiliations:** 1 University of Edinburgh Centre for Genomics and Experimental Medicine, MRC Institute of Genetics and Molecular Medicine, Crewe Road, Edinburgh EH4 2XU, UK and; 2 Now at Centre for Chromosome Biology, School of Natural Sciences, National University of Ireland, Galway, Ireland

## Abstract

Disrupted-In-Schizophrenia 1 (DISC1) is a candidate risk factor for schizophrenia, bipolar disorder and severe recurrent depression. Here, we demonstrate that DISC1 associates robustly with trafficking-protein-Kinesin-binding-1 which is, in turn, known to interact with the outer mitochondrial membrane proteins Miro1/2, linking mitochondria to the kinesin motor for microtubule-based subcellular trafficking. DISC1 also associates with Miro1 and is thus a component of functional mitochondrial transport complexes. Consistent with these observations, in neuronal axons DISC1 promotes specifically anterograde mitochondrial transport. DISC1 thus participates directly in mitochondrial trafficking, which is essential for neural development and neurotransmission. Any factor affecting mitochondrial DISC1 function is hence likely to have deleterious consequences for the brain, potentially contributing to increased risk of psychiatric illness. Intriguingly, therefore, a rare putatively causal human DISC1 sequence variant, 37W, impairs the ability of DISC1 to promote anterograde mitochondrial transport. This is likely related to a number of mitochondrial abnormalities induced by expression of DISC1-37W, which redistributes mitochondrial DISC1 and enhances kinesin mitochondrial association, while also altering protein interactions within the mitochondrial transport complex.

## INTRODUCTION

Disrupted-In-Schizophrenia 1 (DISC1) is a putative risk factor for major mental illness that is involved in critical processes in the developing and adult brain (1–3). DISC1 is expressed in multiple subcellular compartments (1,3), including mitochondria (4–7), where it was recently shown to influence mitochondrial functions including NADH activity, calcium dynamics and monoamine oxidase activity (5). In addition, DISC1-expression levels are reported to affect numbers of motile mitochondria within axons (8). Mitochondrial motility is sensitive to many factors including mitochondrial health (9), and is tightly regulated by various intracellular signaling pathways and other stimuli (10–13). No mechanistic information has yet been provided to explain the reported effect of DISC1 upon mitochondrial motility, nor indeed if this is secondary to effects on mitochondrial function such as those described above (5) or a direct effect upon the mitochondrial trafficking machinery.

Mitochondrial trafficking is critical for brain development and function because mitochondria must be transported around the cell to respond to changing requirements for energy provision and calcium buffering (14). Although mitochondrial transport occurs in most cells, neurons are particularly sensitive to dysfunctional mitochondrial transport, in part because mitochondria must be actively moved along axons and dendrites to reach distant regions such as synapses and growth cones, where demand for energy and calcium buffering is high (14,15). Consequently, any factor leading to suboptimal mitochondrial transport could affect neuronal connectivity and synaptic transmission.

The mitochondrial trafficking process utilizes dynein-mediated retrograde transport and kinesin-mediated anterograde transport. Anterograde mitochondrial trafficking is regulated by multiple proteins, including the Miro/Milton complex. Miro proteins are mitochondrial outer membrane Rho GTPases (16). Milton proteins are kinesin adaptors that bind directly to Miro proteins and thereby recruit Kinesin-1 to mitochondria for their microtubule-based transport around the cell (17). Trafficking-protein-Kinesin-binding-1 (TRAK1) is a mammalian homolog of Milton that is involved in axonal mitochondrial trafficking (18,19). However, while Miro expression is limited to mitochondria, TRAK1 is known to function in trafficking of additional cargoes, including early endosomes and GABA_A_ receptors (20,21).

Here, we investigate mitochondrial DISC1 and describe detrimental effects of a rare DISC1 sequence variant, 37W. This variant has not been identified so far in any unaffected individuals, but has been found in one schizophrenic individual and, within a single Scottish family, it has been found in two patients diagnosed with depression and in one diagnosed with anxiety (22,23). We demonstrate that, through robust association with TRAK1, DISC1 is recruited to mitochondria where it also associates with Miro1. Moreover, DISC1 promotes Kinesin-1 association with mitochondria. Consistent with these observations, DISC1 overexpression increases anterograde axonal mitochondrial trafficking. DISC1-37W elicits several mitochondria-related abnormalities but, unlike DISC1, does not promote anterograde axonal mitochondrial transport. Mitochondrial trafficking defects are thus highlighted as a possible contributory factor in some cases of mental illness.

## RESULTS

### DISC1 associates with TRAK1

**Figure 1.**
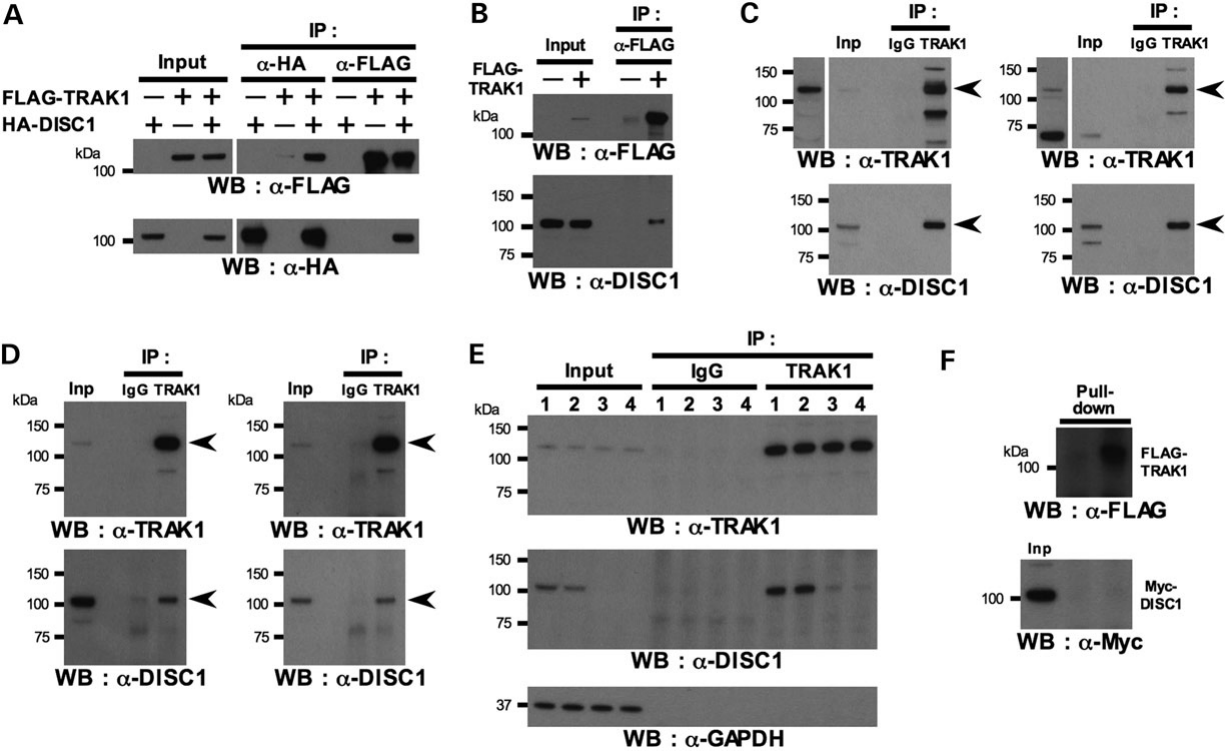
DISC1 interacts with TRAK1. (**A**) COS7 cells were transfected with HA-DISC1 and/or FLAG-TRAK1, or corresponding empty vectors, and subjected to IP using FLAG or HA antibodies. (**B**) HEK293 cells were transfected with FLAG-TRAK1 and subjected to IP using FLAG antibody. (**C**) Non-transfected HEK293 (left) or SH-SY5Y (right) cells were subjected to IP using TRAK1 antibody. Longer exposures of TRAK1-probings of input lysates are shown to the left. Arrowheads indicate full-length TRAK1 or DISC1. (**D**) As (C), but lysates were centrifuged at 100 000*g* to remove detergent-insoluble materials including membrane fractions. Arrowheads indicate full-length TRAK1 or DISC1. (**E**) HEK293 cells were transfected with human DISC1-specific siRNA duplexes. 48 h post-transfection lysates were prepared using 100 000*g* centrifugation and endogenous TRAK1 was subjected to IP. 1: mock transfection (no siRNA), 2: control siRNA, 3: DISC1-siRNA #2, 4: DISC1-siRNA #5. (**F**) *In vitro*-translated FLAG-TRAK1 immobilized to protein G-Sepharose beads was incubated with *in vitro*-translated Myc-DISC1. Inp: input.

We have reported previously that expression of aberrant forms of DISC1 can profoundly affect mitochondrial morphology (24,25), which could be due to effects upon mitochondrial fission, fusion or transport. We therefore carried out a number of speculative co-immunoprecipitation (IP) experiments to determine whether DISC1 associates with factors involved in these processes and demonstrated a strong association between HA-tagged DISC1 and FLAG-tagged TRAK1 in the monkey fibroblast cell line COS7 (Fig. 1A). FLAG-TRAK1 also co-immunoprecipitates endogenous 100 kDa full-length DISC1 from the human embryonic kidney cell line HEK293 (Fig. 1B), while endogenous TRAK1 co-immunoprecipitates endogenous 100 kDa DISC1 from HEK293 cells and the human neuroblastoma cell line SH-SY5Y (Fig. 1C). Even when cell lysates were prepared for IP experiments from HEK293 or SH-SY5Y cells following a more stringent protocol, utilizing high-speed centrifugation for lysate preparation, endogenous TRAK1 still co-immunoprecipitates the 100 kDa form of endogenous human DISC1 from HEK293 and SH-SY5Y cells (Fig. 1D). Moreover, RNA-interference-mediated knockdown of endogenous human DISC1 in HEK293 cells confirmed the identity of the endogenous 100 kDa DISC1 species co-immunoprecipitating with endogenous TRAK1. TRAK1 failed to co-immunoprecipitate this species from high-speed lysates prepared from HEK293 cells treated with short interfering RNAs that knock down DISC1 expression (Fig. 1E). Although we have clearly demonstrated a strong association between DISC1 and TRAK1 in the neuron-like cell line SH-SY5Y, we have not yet confirmed that this association occurs in brain because, in our hands, the two antibodies tested do not detect rodent TRAK1 on blots of brain lysates. Despite the very robust association between TRAK1 and DISC1, both overexpressed and endogenous, we were unable to demonstrate direct binding between these two proteins using *in vitro* synthesized FLAG-TRAK1 and Myc-DISC1 (Fig. 1F). This may indicate that the interaction is indirect. Consistent with this, TRAK1 (otherwise known as OIP106) was pulled out in a large-scale yeast two-hybrid screen using DISC1 and its interactors as bait, where no interaction was observed with DISC1, but potential interactions between TRAK1 and the DISC1 interactors CDC5L and MIP-T3 were identified (26). Alternatively, it is possible that direct interaction between TRAK1 and DISC1 may be mediated by posttranslational modifications that do not occur either in yeast or *in vitro*, thus such a direct interaction would be undetectable using *in vitro* synthesized proteins or yeast two-hybrid screens. Further work will be required to address this point.

**Figure 2.**
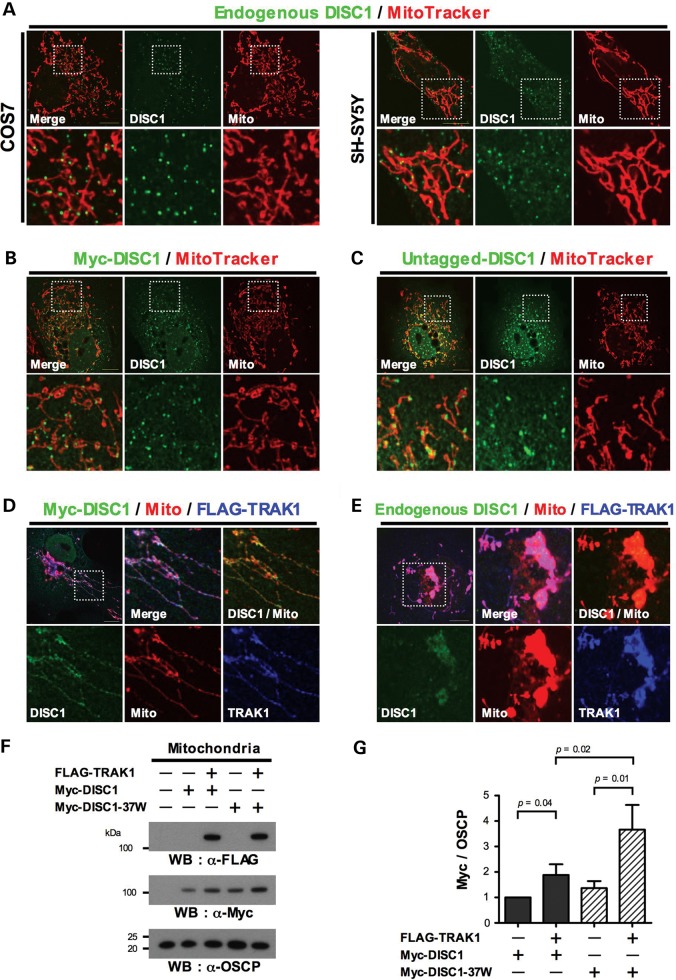
Mitochondrial DISC1 expression. (**A**) Endogenous DISC1 expression in COS7 and SH-SY5Y. (**B**) Myc-DISC1 expression in COS7. (**C**) Untagged exogenous DISC1 expression in COS7 (detected using α-DISC1 antibody at a concentration suitable for detecting overexpressed but not endogenous protein). (**D**) Myc-DISC1 plus FLAG-TRAK1 expression in COS7. (**E**) Endogenous DISC1 plus FLAG-TRAK1 expression in COS7. (**F**) Mitochondria were isolated from COS7 cells transfected with Myc-DISC1 or Myc-DISC1-37W with or without FLAG-TRAK1, immunoblotted and probed with antibodies specific for Myc, FLAG or the mitochondrial marker OSPC. (**G**) Quantification of data obtained in (F). *n* = 6 independent transfections. Perinuclear mitochondrial clustering induced by TRAK1 overexpression (Supplementary Material, Fig. S1) is clearly visible in (D) and (E). Except where indicated α-DISC1 antibody was used to detect endogenous DISC1. Error bars represent standard error of the mean (SEM). Statistical analysis was performed using the one-tailed (paired) Student's *t*-test. Outlined areas are shown expanded. Scale bars; 10 μm.

In COS7 and SH-SY5Y cells, endogenous mitochondrial DISC1 is distributed as puncta, generally located around the mitochondrial periphery (Fig. 2A). Myc-DISC1 (Fig. 2B) and exogenous untagged DISC1 (Fig. 2C) distribute similarly in COS7 cells. Although TRAK1 expression is not limited to mitochondria (20), using immunofluorescence FLAG-TRAK1 appears to be almost exclusively mitochondrial in COS7 cells (Supplementary Material, Fig. S1A). Moreover, when FLAG-TRAK1 is expressed in COS7 cells their mitochondria appear either normal, swollen or in the majority of cells, partially or completely clustered (Supplementary Material, Fig. S1A), as has been reported previously for TRAK1 overexpression in HEK293 cells (18). In COS7 cells co-transfected with Myc-DISC1 plus FLAG-TRAK1, the punctate mitochondrial Myc-DISC1 distribution decreases and mitochondrial DISC1 expression becomes more pronounced and homogeneous (Fig. 2D). FLAG-TRAK1 similarly redistributes endogenous DISC1 in COS7 cells (Fig. 2E), indicating that TRAK1 influences the mitochondrial distribution of DISC1 and indeed, may recruit DISC1 to mitochondria. This recruitment was confirmed by isolating mitochondria from transfected COS7 cells. In the presence of FLAG-TRAK1, mitochondrial Myc-DISC1 expression increases by ∼1.9-fold (*P* = 0.04, Fig. 2F and G).

**Figure 3.**
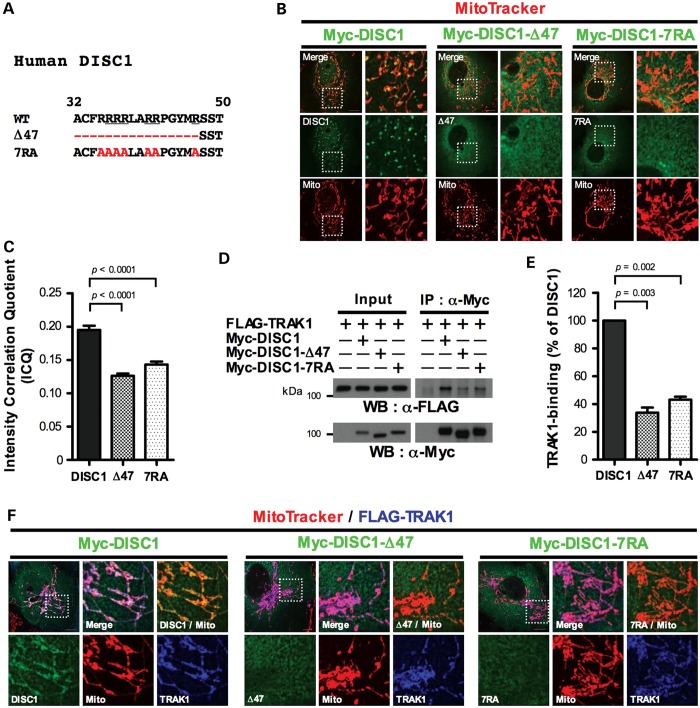
The arginine-rich sequence motif mediates TRAK1 interaction and mitochondrial DISC1 expression. (**A**) Sequence alignment of the arginine-rich motif in wild-type human DISC1 and mutant forms, Δ47 and 7RA. Evolutionarily conserved arginine residues and mutated amino acids are underlined or highlighted in red, respectively. (**B**) Expression of Myc-DISC1, Myc-DISC1-Δ47 or Myc-DISC1-7RA in COS7. (**C**) Intensity correlation analysis of mitochondrial DISC1 expression. *n* = 3 independent transfections, 10 cells counted/experiment. (**D**) COS7 cells expressing FLAG-TRAK1 plus Myc-DISC1, Myc-DISC1-Δ47 or Myc-DISC1-7RA were subjected to IP using Myc antibody. Cells that did not receive TRAK1 or DISC1 were instead transfected with corresponding empty vector. (**E**) Quantification of data obtained in (D). *n* = 3 independent transfections. (**F**) Expression of FLAG-TRAK1 plus Myc-DISC1, Myc-DISC1-Δ47 or Myc-DISC1-7RA in COS7. Perinuclear mitochondrial clustering induced by TRAK1 overexpression (Supplementary Material, Fig. S1) is clearly visible in (F). Error bars represent SEM. Statistical analysis was performed using the two-tailed Student's *t*-test, unpaired only for intensity correlation analysis. Outlined areas are shown expanded. Scale bars: 10 μm.

The DISC1 head domain contains a conserved arginine-rich sequence motif (27) (Fig. 3A) that acts as a nuclear localization signal (28). However, we show that this motif also determines mitochondrial DISC1 expression using confocal imaging combined with intensity correlation analysis, which provides a measure of protein co-localization within cells. Myc-DISC1 clearly associates with mitochondria in a punctate pattern (Fig. 3B). In contrast, upon deletion of the N-terminal 47 amino acids that encompass the arginine-rich motif (Fig. 3A), the punctate mitochondrial distribution of Myc-DISC1 is virtually abolished (Fig. 3B) and co-localization with mitochondria is reduced (*P* < 0.0001, Fig. 3C). Similarly, when all seven arginine residues in the motif are replaced with alanine (Fig. 3A), the punctate distribution of Myc-DISC1 is ablated (Fig. 3B) and co-localization with mitochondria decreases (*P* < 0.0001, Fig. 3C).

Since TRAK1 regulates the mitochondrial distribution of DISC1, we used the mutant forms of DISC1 that ablate its punctate mitochondrial distribution to determine whether the arginine-rich motif mediates DISC1/TRAK1 association. Deletion of amino acids 1–47 (Δ47) or mutation of the seven arginine residues to alanine (7RA) significantly reduces Myc-DISC1/FLAG-TRAK1 co-IP from COS7 cells (*P* = 0.003 for Δ47, *P* = 0.002 for 7RA, Fig. 3D and E), and decreases their co-localization, with FLAG-TRAK1 expression remaining strongly mitochondrial, while Myc-DISC1 becomes cytoplasmic (Fig. 3F). The association between DISC1 and TRAK1 is therefore mediated, perhaps via an intermediary protein or posttranslational modification, by the arginine-rich motif, and the arginine-rich motif-dependent punctate mitochondrial distribution adopted by DISC1 is likely due to association with TRAK1.

### Effects of a rare DISC1 sequence variant, R37W

**Figure 4.**
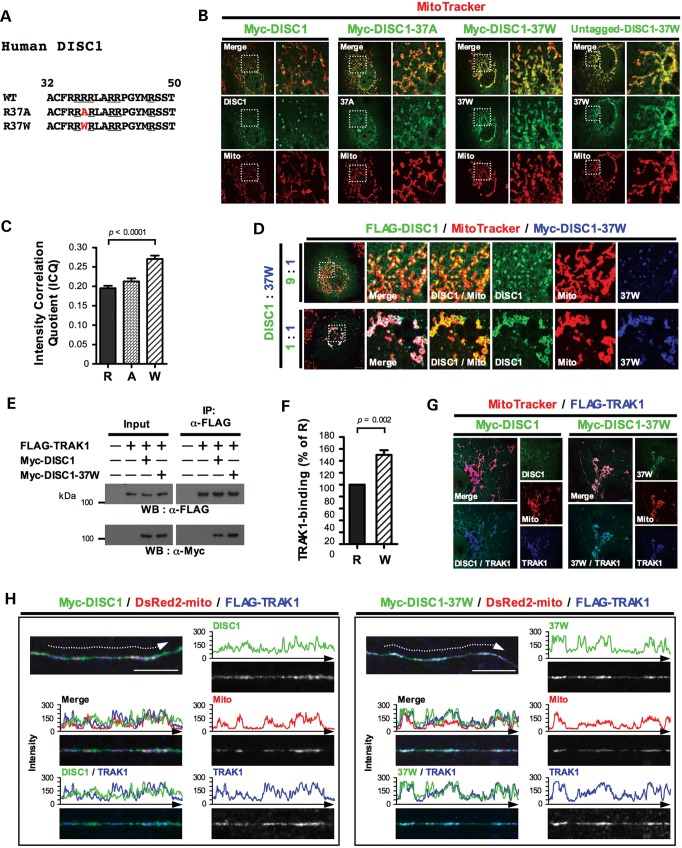
Effects of the 37W variant. (**A**) Sequence alignment of the arginine-rich motif in wild-type human DISC1, and DISC1-37A or DISC1-37W. Evolutionarily conserved arginine residues and mutated amino acids are underlined or highlighted in red, respectively. (**B**) Representative images showing expression of Myc-DISC1, Myc-DISC1-37A, Myc-DISC1-37W or untagged DISC1-37W in COS7. (**C**) Intensity correlation analysis of mitochondrial DISC1 expression. R: DISC1, A: DISC1-37A, W: DISC1-37W. *n* = 3 independent transfections, 10 cells counted/experiment. (**D**) COS7 cells were co-transfected with FLAG-DISC1 plus Myc-DISC1-37W in different proportions, using the same amount of total plasmid DNA per transfection. (**E**) COS7 cells expressing FLAG-TRAK1 plus Myc-DISC1 or Myc-DISC1-37W, or corresponding empty vectors, were subjected to IP using FLAG antibody. (**F**) Quantification of data obtained in (E). R: DISC1, W: DISC1-37W. *n* = 6 independent transfections. (**G**) FLAG-TRAK1 plus Myc-DISC1 or Myc-DISC1-37W expression in COS7. (**H**) Mouse hippocampal neurons (DIV 19) were transfected with DsRed2-mito and FLAG-TRAK1 plus Myc-DISC1 or Myc-DISC1-37W. Staining was performed 1 day post-transfection. Panels show straightened axons and the signal intensities of pixels from each color scanned along dotted arrows using ImageJ software. Perinuclear mitochondrial clustering induced by TRAK1 or DISC1-37W overexpression (Supplementary Material, Fig. S1) is clearly visible in (D) (lower) and (G). Error bars represent SEM. Statistical analysis was performed using the two-tailed Student's *t*-test, unpaired only for intensity correlation analysis. Outlined areas are shown expanded. Scale bars: 10 μm.

Arginine 37 is located within the arginine-rich motif (Fig. 4A). Intriguingly, its mutation to tryptophan (37W) alters the distribution of mitochondrial DISC1, for both Myc-tagged and -untagged forms (Fig. 4B); due to the 37W variant, the mitochondrial DISC1 distribution loses its punctate appearance, becoming more homogeneous. This change is similar to that induced by FLAG-TRAK1 overexpression (Fig. 2D). Intensity correlation analysis confirmed the more homogeneous mitochondrial appearance of Myc-DISC1-37W versus Myc-DISC1 (Fig. 4C, *P* < 0.0001). Substituting an alanine for arginine at position 37 (37A) does not significantly alter the mitochondrial association (Fig. 4B and C), suggesting that tryptophan substitution is the main factor responsible for the changed mitochondrial distribution of DISC1. We isolated mitochondria from transfected COS7 cells to investigate whether the altered mitochondrial distribution pattern of DISC1-37W is due to increased mitochondrial targeting, but found that mitochondrial DISC1 and DISC1-37W are expressed at similar levels (Fig. 2F and G). The 37W variant thus affects DISC1 distribution pattern, but not total expression, at mitochondria.

DISC1-37W additionally induces perinuclear mitochondrial clustering in ∼50% of transfected COS7 cells (Supplementary Material, Fig. S1B and C). Again this phenotype is remarkably similar to that induced in most transfected COS7 cells by FLAG-TRAK1 overexpression (Supplementary Material, Fig. S1).

We recently demonstrated that, in transfected cell lines, DISC1-37W disrupts nuclear targeting of wild-type DISC1 in a dominant-negative fashion (29). Consistent with this, when COS7 cells are co-transfected with Myc-DISC1-37W plus FLAG-DISC1, both DISC1 species co-localize and adopt the distinctive pattern of mitochondrial distribution induced by DISC1-37W (Fig. 4D). This effect of DISC1-37W upon the distribution of DISC1 occurs at a 1:1 ratio and is still evident when FLAG-DISC1 is present in excess. This predicts a dominant effect at the cellular level in 37W carriers.

The location of 37W within the TRAK1 association site suggested that this variant may influence DISC1/TRAK1 association. Immunoprecipitation experiments revealed 50% increased association of Myc-DISC1-37W with FLAG-TRAK1 in COS7 cells compared to Myc-DISC1 (*P* = 0.002, Fig. 4E and F). When high-speed lysates are used for these experiments the increased association is again apparent (Supplementary Material, Fig. S2). This increased association is also detectable by immunofluorescence whereby Myc-DISC1-37W exhibits increased co-localization with FLAG-TRAK1 at mitochondria in COS7 cells (Fig. 4G). FLAG-TRAK1 expression recruits Myc-DISC1-37W to mitochondria (*P* = 0.01, Fig. 2F and G) and, consistent with the increased DISC1/TRAK1 association induced by the 37W variant, Myc-DISC1-37W is recruited ∼1.9-fold more than Myc-DISC1 to mitochondria in the presence of FLAG-TRAK1 (*P* = 0.02, Fig. 2F and G). In axons of primary mouse hippocampal neurons co-transfected with FLAG-TRAK1, the association of DISC1 with mitochondria, and with TRAK1, also becomes more distinct when co-transfected with Myc-DISC1-37W (Fig. 4H). Taken together, these observations demonstrate that the surprisingly similar effects of DISC1-37W overexpression to those of TRAK1 overexpression upon the mitochondrial distribution of DISC1, i.e. the change from punctate to homogeneous mitochondrial association, are likely attributable, at least in part, to increased association of DISC1-37W with TRAK1.

The reason for the 37W-mediated increased association with TRAK1 is unknown, but it is possible that R37 either directly mediates contact with TRAK1 or an intermediary protein, or that the non-conservative change to tryptophan may indirectly alter the conformation or accessibility of the binding site for TRAK1 or an intermediary protein; it is reasonable to assume that R37 resides within a hydrophilic segment because it is located within a tetra-arginine stretch, a known nuclear localization motif (28). While the positive charge of the arginine-rich motif could be required for the formation of ionic bonds with oppositely charged residues on another protein, the presence of the aromatic tryptophan side chain may distort the conformation of the hydrophilic segment surrounding residue 37 and make binding more favorable. Alternatively, the introduction of hydrophobicity could alter the association/dissociation constants for binding in comparison to the electrostatic interaction afforded by DISC1.

### DISC1-37W promotes Kinesin-1 association with mitochondria

**Figure 5.**
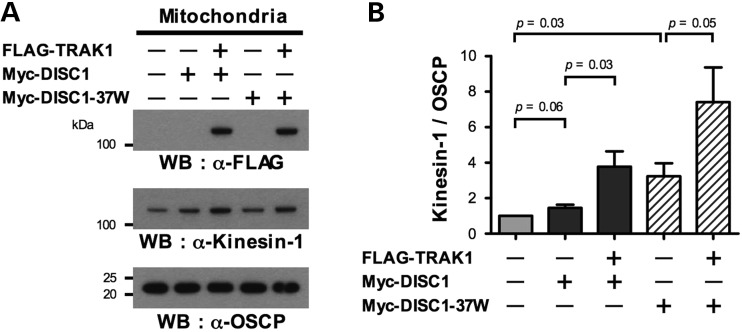
DISC1 promotes Kinesin-1 association with mitochondria. (**A**) Mitochondria were isolated from COS7 cells transfected with Myc-DISC1 or Myc-DISC1-37W with or without FLAG-TRAK1, immunoblotted and probed with antibodies specific for Kinesin-1, FLAG or the mitochondrial marker OSPC. (**B**) Quantification of Kinesin-1/OSCP ratios using the data obtained in (A). *n* = 6 independent transfections. This is the same experimental series as referred to in Figure 2F and G. Error bars represent SEM. Statistical analysis was performed using the (paired) two-tailed Student's *t*-test.

The increased association of DISC1-37W with TRAK1 suggested that 37W may induce additional altered associations within the mitochondrial transport complex, where TRAK1 interacts directly with Miro and Kinesin-1, and indirectly with alpha-tubulin. Expression of Myc-DISC1 or Myc-DISC1-37W has no effect upon the association of endogenous alpha-tubulin or Kinesin-1 with FLAG-TRAK1 by IP (Supplementary Material, Fig. S3). However, using subcellular fractionation we found that DISC1 overexpression shows a trend towards increasing the association of endogenous Kinesin-1 with isolated mitochondria; Myc-DISC1 increases the association by 1.4-fold on average (*P* = 0.06, Fig. 5A and B). Myc-DISC1-37W has a stronger effect, increasing the association by an average 3.2-fold (*P* = 0.03, Fig. 5A and B). Thus, while we found no evidence that DISC1 modulates the association of Kinesin-1 with TRAK1, there is an effect upon mitochondrial Kinesin-1 recruitment that is augmented by the 37W variant.

DISC1 is reported to interact directly with Kinesin-1 (KIF5A) (30), consequently it is possible that the DISC1-dependent recruitment of Kinesin-1 (an antibody to KIF5B was used in our experiments) to mitochondria occurs in part because Kinesin-1 accompanies the overexpressed DISC1. However, it seems unlikely that the 37W variant augments mitochondrial Kinesin-1 expression simply by increasing DISC1/Kinesin-1 interaction, because the known KIF5A binding domain does not include the region of DISC1 encompassing R37 (30). The detailed mechanism by which the 37W variant enhances Kinesin-1 recruitment will therefore be an interesting point for future investigations.

It is worth noting that FLAG-TRAK1 co-transfection with Myc-DISC1 or Myc-DISC1-37W, apparently enhances the recruitment of Kinesin-1 to mitochondria still further. This effect was observed for DISC1 (*P* = 0.03, Fig. 5A and B), and a similar trend was observed for DISC1-37W (*P* = 0.05, Fig. 5A and B). This is consistent with the role of TRAK1 as a kinesin adaptor protein, but may also be related to its recruitment of DISC1 to mitochondria.

### DISC1 associates with Miro1

**Figure 6.**
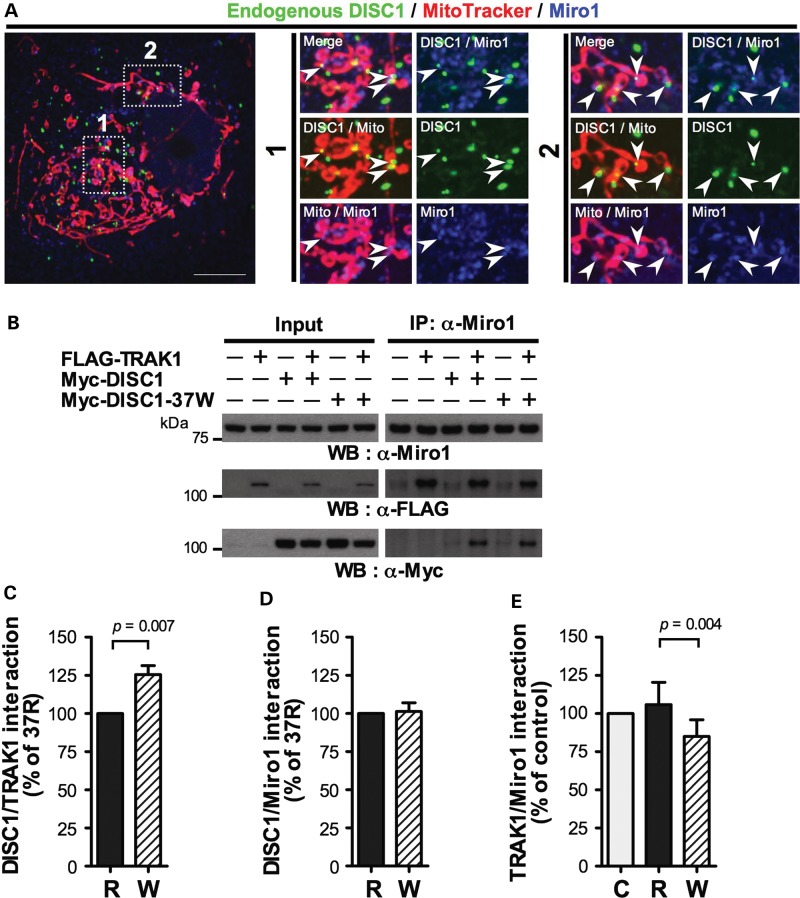
DISC1 interacts with Miro1, effects of 37W. (**A**) Endogenous DISC1 and Miro1 expression in COS7. Outlined areas are shown expanded. Arrowheads indicate examples of DISC1/Miro1 overlap. Scale bar: 10 μm. (**B**) COS7 cells were co-transfected with FLAG-TRAK1 plus either Myc-DISC1 or Myc-DISC1-37W, or corresponding empty vectors and subjected to IP using Miro1 antibody. (**C**–**E**) Quantification of data obtained in (B). C: empty vector, R: DISC1, W: DISC1-37W. *n* = 6 independent transfections. Error bars represent SEM. Statistical analysis was carried out using the (paired) two-tailed Student's *t*-test.

We next investigated Miro, and first determined whether DISC1 associates with Miro1. In COS7 cells many, but not all, mitochondria-associated endogenous DISC1 puncta overlap with endogenous Miro1 (Fig. 6A). Moreover, Myc-DISC1 co-immunoprecipitates with endogenous Miro1 (Fig. 6B) although co-transfection of FLAG-TRAK1 increases Myc-DISC1/Miro1 association substantially (Fig. 6B). DISC1 association with Miro1 is thus apparently facilitated by TRAK1. As expected, FLAG-TRAK1 also co-immunoprecipitates with endogenous Miro1 (Fig. 6B). The increased association of DISC1-37W with TRAK1 (Fig. 4E and F) also occurs in Miro1 immunoprecipitates (*P* = 0.007, Fig. 6C). Unexpectedly, however, 37W does not affect the amount of Myc-DISC1 co-immunoprecipitating with Miro1 (Fig. 6D). Quantification of FLAG-TRAK1 co-IP with Miro1 demonstrated that these observations are reconciled by 20% decreased FLAG-TRAK1 association with Miro1 in cells expressing Myc-DISC1-37W compared with Myc-DISC1 (*P* = 0.004, Fig. 6E). Because Miro1 is embedded in the outer mitochondrial membrane and acts as an anchor for TRAK1, this reduced FLAG-TRAK1/Miro1 interaction might be predicted to affect the coupling between mitochondria and TRAK1. Myc-DISC1-37W does not, however, detectably decrease mitochondrial localization of FLAG-TRAK1 (Fig. 4G and H). We thus hypothesize that expressing DISC1-37W dysregulates coupling of mitochondria to the transport machinery, rather than severing the connection. Consistent with this suggestion, human Milton proteins can associate with mitochondria independent of Miro (31). In contrast to Myc-DISC1-37W, Myc-DISC1 does not detectably alter the average association between FLAG-TRAK1 and Miro1 across several experiments (Fig. 6E).

### DISC1 promotes anterograde mitochondrial trafficking

**Figure 7.**
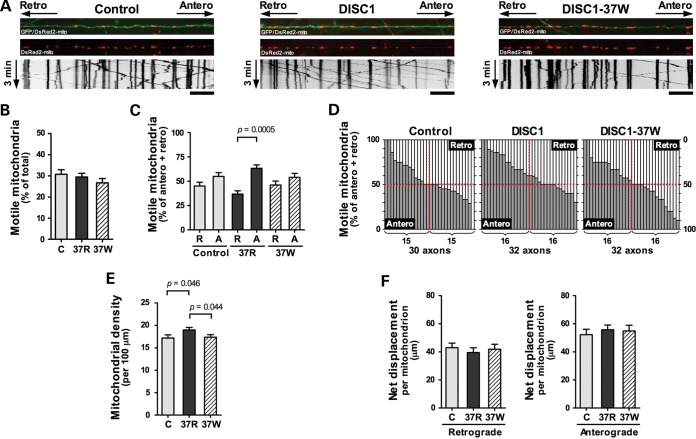
DISC1 promotes anterograde mitochondrial transport. Hippocampal neurons (DIV 5–9) were transfected with GFP, DsRed2-mito and either empty vector, FLAG-DISC1 or FLAG-DISC1-37W. Time-lapse imaging was performed 1 day post-transfection. (**A**) Representative images of axons and kymographs. Scale bars: 20 μm. (**B**) Mitochondrial motility expressed as percentage of total mitochondria within axons. (**C**) Motile mitochondria moving in the anterograde, A, or retrograde, R, direction expressed as percentage of anterograde plus retrograde mitochondria per axon. (**D**) Representation of anterograde (dark gray) versus retrograde (light gray) movement within each axon. Each bar represents an individual axon. (**E**) Average mitochondrial density within axons. (**F**) Average net mitochondrial displacement. *n* = 5 independent experiments. Error bars represent SEM. Statistical analysis was carried out using the two-tailed Student's *t*-test, paired only for analysis of retrograde versus anterograde mitochondria within axons.

Because DISC1 modulates Kinesin-1 association with mitochondria and is a component of the mitochondrial trafficking machinery, we next examined mitochondrial transport (Fig. 7A and Table 1). We did not detect any effect of exogenous DISC1 upon total axonal mitochondrial movement (Fig. 7B). However, FLAG-DISC1 overexpression clearly promotes anterograde mitochondrial movement. Thus, while there is no change in the number of motile mitochondria per axon in neurons transfected with DISC1, more mitochondria are moving in the anterograde direction rather than the retrograde direction per axon (*P* = 0.0005 for DISC1, not significant for empty vector, Fig. 7C and D). Consistent with this, the axonal density of mitochondria was increased in neurons transfected with DISC1 (*P* = 0.046, Fig. 7E). There was no effect of DISC1 upon mitochondrial displacement, a surrogate measure of velocity (Fig. 7F). It is possible that the enhanced anterograde mitochondrial trafficking that occurs in axons in response to DISC1 overexpression is related to increased mitochondrial expression of the anterograde motor Kinesin-1 (Fig. 5), albeit we only observed a statistical trend towards DISC1-induced recruitment of Kinesin-1 (Fig. 5A and B).

**Table 1.** Effect of DISC1 expression upon axonal mitochondrial motility

	Axons	Mitochondria total, anterograde, retrograde	Average % motility	Average anterograde mitochondria (% of motile)	Average retrograde mitochondria (% of motile)
Empty vector	30	791, 129, 107	30.8 ± 2.1	51.8 ± 3.6	42.3 ± 3.5
DISC1	32	949, 175, 108	29.5 ± 1.7	61.3 ± 3.5	35.5 ± 3.4
DISC1-37W	32	924, 127, 117	26.7 ± 2.1	52.2 ± 4.1	44.5 ± 4.0

Average values are represented ± SEM of all axonal values. Motile mitochondria that were not classed as anterograde or retrograde were either oscillating or their overall direction of movement could not be determined.

A previous study (8) reported that DISC1 overexpression promotes total mitochondrial motility (with no investigation of directional movement). Thus, while both studies demonstrate that DISC1 promotes mitochondrial trafficking, they are not in agreement about the effect upon total motility. This discrepancy may be due to differing experimental protocols: our assays were carried out in different media, in mouse as opposed to rat, and at 37°C as opposed to 35°C, a temperature difference that affects mitochondrial motility (32) and could therefore influence motility responses to DISC1 overexpression. Moreover, our measurements were taken one day post-transfection as opposed to two: the effect of DISC1 upon motility may progress with exposure time.

DISC1-37W had no significant effect upon total mitochondrial motility (Fig. 7B). Moreover, unlike DISC1, DISC1-37W does not promote anterograde movement (Fig. 7C and D), nor does it increase total mitochondrial density (Fig. 7E). The 37W variant thus apparently impairs the ability of DISC1 to stimulate anterograde mitochondrial transport. These observations may appear at odds with our suggestion that DISC1-dependent Kinesin-1 recruitment promotes anterograde mitochondrial transport because DISC1-37W also enhances mitochondrial Kinesin-1 expression, even more so than DISC1 (Fig. 5). However, DISC1-37W is expressed aberrantly at mitochondria where it induces altered interactions within the mitochondrial transport complex (Fig. 4). These, and other as yet unknown consequences of the 37W variant, may counteract the effect of Kinesin-1 recruitment. Overall then, DISC1 appears to be a novel regulator of the direction of mitochondrial movement, and this role is compromised by the rare 37W variant.

## DISCUSSION

In this study, we have clearly demonstrated robust association of DISC1 with mitochondrial transport complexes containing TRAK1 and Miro1. These complexes link mitochondria to kinesin and thence to the microtubule tracks that are utilized for mobilizing mitochondria around the mammalian cell (18,33). This places DISC1 in a position to participate directly in mitochondrial trafficking even though there may be no direct interaction between DISC1 and TRAK1 or Miro1. This inclusion of DISC1 in mitochondrial transport complexes in human cell lines is strikingly consistent with our demonstration that DISC1 promotes anterograde axonal trafficking within neurons, possibly in part via recruitment of Kinesin-1 to mitochondria. It is thus likely that DISC1 directly influences directional mitochondrial movement by complexing with TRAK1 and Miro1 in neurons. In support of DISC1 associating with the neuronal mitochondrial transport machinery, we have shown that DISC1 co-localizes with TRAK1 in neurons. Moreover, the DISC1 arginine-rich motif required for TRAK1 binding is conserved in mouse, apart from a conservative substitution of glutamine for the first arginine (27). However, due to antibody limitations, we have not confirmed that the robust DISC1/TRAK1 association we demonstrated using endogenous proteins in human cell lines does indeed occur in mouse neurons. Consequently, it is possible that TRAK1 and DISC1 do not complex in neurons, either because their expression does not overlap or because DISC1 associates with other protein complexes, and that DISC1 performs TRAK1-independent functions that indirectly influence directional mitochondrial motility.

Because DISC1 regulates the direction of mitochondrial motion, we speculate that it mediates the recruitment of mitochondria to distant regions of the neuron with high demand for energy provision or calcium buffering that occurs in response to signals such as serotonin or dopamine (10,11). In this model, DISC1 dysfunction would be predicted to hinder the ability of neurons to respond to such signals by transporting mitochondria to where they are most needed. Inheritance of the 37W variant is thus likely to be deleterious for brain development and function due to effects upon the mitochondrial transport machinery and impaired regulation of the direction of mitochondrial trafficking, in addition to its previously described effects upon nuclear DISC1 expression and gene transcription (29). Consistent with our observations, the 37W variant may influence risk of psychiatric illness because it has only been observed in psychiatric patients, but not in well over 5000 controls: it was first identified in a single schizophrenic individual, of 288 schizophrenic individuals screened, but found to be absent from 288 controls and then a further 5000 controls (22). We subsequently identified the same variant in a proband diagnosed with major depressive disorder, from a screen of 653 Scottish cases of schizophrenia, bipolar disorder or depression and 889 controls. Two of the proband's family members, one affected with major depressive disorder the other with generalized anxiety, are also carriers, but not a further family member affected with bipolar disorder or two unaffected relatives (23). In addition to the effects of the 37W variant, DISC1 is strongly implicated as a risk factor for schizophrenia, bipolar disorder and recurrent depression because the DISC1 gene is directly disrupted by a chromosomal t(1;11) translocation that causes these disorders in a large Scottish family (34,35). This translocation causes DISC1 haploinsufficiency (36) and, intriguingly, individuals carrying the t(1;11) translocation express transcripts encoding aberrant DISC1 species that induce mitochondrial abnormalities (24). Both these effects of the translocation may be detrimental to mitochondrial trafficking via dysregulation of the mitochondrial transport machinery. Altogether therefore, we propose that mitochondrial trafficking dysfunction may contribute to risk of mental illness in individuals carrying deleterious variants or mutations affecting DISC1 expression and/or function.

Several neurodegenerative disorders including Parkinson's, Huntington's and Alzheimer's diseases also exhibit mitochondrial dysfunction (14). In particular, autosomal recessive Parkinson's disease is caused by mutations in PTEN-induced putative Kinase 1 (PINK1) and Parkin (37). The kinase PINK1 acts upstream of the E3 ubiquitin ligase Parkin, phosphorylating Miro and targeting it for Parkin-dependent degradation (38). Removal of Miro detaches kinesin from the mitochondria, blocks their motility and thus sequesters damaged mitochondria prior to clearance (38). The mutations in PINK1 and Parkin interfere with this process, leading to accumulation of damaged mitochondria. This pathway therefore merits further examination to better understand how, and with what consequences, DISC1 affects mitochondrial function in the developing and adult brain, and if/how this influences risk and/or progression of mental illness, or indeed Parkinson's disease and other neurodegenerative diseases where mitochondrial trafficking is defective. It is important to note, however, that the disorders in which DISC1 is most strongly implicated, namely schizophrenia, bipolar disorder and recurrent major depression, are not characterized by extensive neurodegeneration.

Finally, it is worth pointing out that, while this study has focused upon mitochondrial trafficking, DISC1 also functions in synaptic vesicle transport (39). Moreover, TRAK1 regulates motility of cargoes other than mitochondria. TRAK1 associates with, and regulates trafficking of endosomes to lysosomes, an essential step in degradation of cell surface molecules, such as receptors (20). TRAK1 additionally associates with GABA_A_ receptors and may also regulate their trafficking (21). Altogether then, there is potential for DISC1 to have a general role in regulation of intracellular transport processes that are critical for brain function.

## MATERIALS AND METHODS

### Plasmids

Full-length human DISC1 carrying an N-terminal Myc or HA epitope tag was expressed from pcDNA3.1. The pcDNA3.1 Myc-DISC1-expression construct was used as a template to generate the Myc-DISC1-7RA, Myc-DISC1-37W and Myc-DISC1-37A mutants, with the following primer sets by using the QuikChange II site-directed mutagenesis kit (Stratagene) or conventional PCR methods.

7RA

Forward primer (5′–3′):

GCGGCGGCGGCGCTGGCAGCGGCGCCGGGCTACATGGCAAGCTCGACAGGGCC

Reverse primer (5′–3′):

TGCCATGTAGCCCGGCGCCGCTGCCAGCGCCGCCGCCGCAAAGCACGCTGCAGG

37W

Forward primer (5′–3′):

GCGTGCTTTCGGAGGTGGCGGCTGGCACGGAGGC

Reverse primer (5′–3′):

GCCTCCGTGCCAGCCGCCACCTCCGAAAGCACGC

37A

Forward primer (5′–3′):

GCAGCGTGCTTTCGGAGGGCGCGGCTGGCACGGAG

Reverse primer (5′–3′):

CTCCGTGCCAGCCGCGCCCTCCGAAAGCACGCTGC

pcDNA3.1 Myc-DISC1-Δ47 encodes N-terminally Myc-tagged human DISC1, corresponding to aa 48–854. pcDNA3.1 constructs expressing untagged DISC1 were generated using pcDNA Myc-DISC1 constructs as templates. pcDNA3.1 FLAG-DISC1, encoding N-terminally FLAG-tagged human DISC1 was used for generating the data in Figure 4D. pcDNA4/TO N-terminally FLAG- or Myc-tagged DISC1- or DISC-37W-expression vectors (29) were used for live cell imaging, immunostaining of neurons, mitochondrial fractionation experiments and some IPs. Human TRAK1 cDNA (KIAA1042) was purchased from the Kazusa DNA Research Institute in Japan (http://www.kazusa.or.jp). pcDNA4/TO FLAG-TRAK1 and pcDNA3.1 FLAG-TRAK1 encode N-terminally FLAG-tagged TRAK1 (aa 2–953).

### Cell culture and transfection

COS7, HEK293 and SH-SY5Y cells were maintained in Dulbecco's modified Eagle medium (Invitrogen) containing 10% fetal bovine serum at 37°C in a 5% CO_2_ humidified atmosphere. Hippocampal neuron cultures were prepared from C57BL/6 mouse fetuses (E17-18) as described previously (40), grown on coverslips coated with poly-d-lysine and maintained in neurobasal medium supplemented with 2% B-27 and 2 mm Glutamax (all from Invitrogen) at 37°C with 5% CO_2_. Transfection of cultured cells and hippocampal neurons was conducted using Lipofectamine 2000 (Invitrogen) according to the manufacturer's instruction. Twenty to twenty four hour post-transfection, cells were harvested for mitochondria isolation, immunofluorescence or IP experiments.

### Live imaging of mitochondria in axons of hippocampal neurons

u-Dishes (Ibidi) were coated with poly-d-lysine and then two Culture-Inserts (Ibidi) were placed in an u-Dish to allow us to analyze up to four different samples at a time in the same dish. 5 × 10^4^ cells were plated in each well in neurobasal medium containing B-27 supplement and 2 mm Glutamax without phenol red (Invitrogen). Transfection of hippocampal neurons was conducted between days *in vitro* (DIV) 5–9 using Lipofectamine 2000. pcDNA4/TO empty vector, pcDNA4/TO FLAG-DISC1 or pcDNA4/TO FLAG-DISC1-37W was introduced into hippocampal neurons with pDsRed2-mito and pMax-GFP. Expression plasmids (pcDNA4/TO, pDsRed2-mito and pMax-GFP) were transfected in a 4:1:1 ratio. One hour after incubation with DNA/lipofectamine complexes, neurons were gently washed with neurobasal medium once. Culture-Inserts were then removed and neuron cultures were maintained in neurobasal medium for 20–24 h until live imaging (DIV 6–10). Imaging of mitochondrial movement in axons was performed at 37°C in 5% CO_2_ in a climate-controlled chamber. Axonal sections which were selected for recording (Supplementary Material, Fig. S4A) [average lengths of the axonal sections (control *N* = 30; 155.7 μm ± 5.2, DISC1 *N* = 32; 156.2 μm ± 3.7, DISC1-37W *N* = 32; 163.2 μm ± 5.1)] were at least 100 μm away from the cell soma [average distance from the cell soma (control *N* = 30; 185.6 μm ± 14.8, DISC1 *N* = 32; 170.9 μm ± 13.3, DISC1-37W *N* = 32; 200.5 μm ± 16.7)]. All images were acquired with a Photometrics Evolve EMCCD camera on a Nikon C1SI/TiE microscope equipped with a Plan-Fluor 40×/0.75 OFN25 DIC objective plus an additional ×1.5 optical lens (×60 total). Images of axonal mitochondria were taken every 2 s for 3 min to generate movies (91 stacks total). Kymographs were generated from movies with NIS-Elements software (Nikon). Black/white colors in kymographs were inverted for clarity using Adobe Photoshop. Motile mitochondria were defined as those that moved more than 3 μm in 3 min, based on a threshold used previously (8). Curved axons were straightened using ImageJ software. Transfection and live imaging were conducted in a blinded fashion. After live imaging, neurons were fixed in 4% formaldehyde and triple-stained with anti-human DISC1 C-ter pAb (α-DISC1) to confirm DISC1-expression in GFP and DsRed-positive neurons (Supplementary Material, Fig. S4B).

### *In vitro*-binding assay

Myc-DISC1 or FLAG-TRAK1 was synthesized *in vitro* using the TNT T7 Coupled Reticulocyte Lysate System (Promega). *In vitro*-translated FLAG-TRAK1 was incubated with anti-FLAG mAb and then coupled to protein G-Sepharose beads in binding buffer [1% Triton X-100, 50 mm Tris–HCl (pH 7.5), 150 mm NaCl, Protease Inhibitor Cocktail (Roche)]. After extensive wash with binding buffer, FLAG-TRAK1-coupled beads were further incubated with *in vitro*-translated Myc-DISC1 in binding buffer overnight at 4°C. The beads were washed with binding buffer three times and with 50 mm Tris–HCl (pH 7.5) once, followed by western blot analyses.

### RNA interference

The following siRNA sequences against exon 2 or 13 of human DISC1 were selected.

Silence select siRNA DISC1 #2 (exon 13)

Sense: (5′–3′): GGAUUUGAGAAUAGUUUCAtt

Antisense: (5′–3′): UGAAACUAUUCUCAAAUCCtt

Silencer select siRNA DISC1 #5 (exon 2)

Sense: (5′–3′): GCGUGACAUGCAUUCUUUAtt

Antisense: (5′–3′): UAAAGAAUGCAUGUCACGCtt

Sense or antisense oligonucleotides were synthesized, HPLC-purified and annealed by Ambion. Silencer Select Negative Control siRNA was also purchased from Ambion. HEK293 cells were transfected with Control or DISC1 siRNA at a final concentration of 50 nm using Lipofectamine 2000. Transfected cells were then incubated for 48 h prior to harvesting for IP experiments.

### Immunoprecipitation

COS7, HEK293 or SH-SY5Y cells were lysed in IP buffer [1% Triton X-100, 50 mm Tris–HCl (pH 7.5), 150 mm NaCl, Protease Inhibitor Cocktail (Roche), Phosphatase Inhibitor Cocktail Set II (Calbiochem)] and insoluble materials were removed by centrifugation at 17 000*g* or 100 000*g* (Beckman TLA 100.3) for 30 min at 4°C. Cell lysates were precleared with protein G-Sepharose beads (Sigma) alone for 1 h at 4°C, and then incubated with anti-Myc mAb, anti-HA pAb, anti-FLAG M2 mAb or anti-TRAK1 pAb overnight at 4°C. Protein G-Sepharose beads were added to the lysates and further incubated to capture immunocomplexes for 2 h. The beads were washed with IP buffer three to four times and with 50 mm Tris–HCl (pH 7.5) once followed by western blot analyses. For quantification of some protein–protein interactions (DISC1/TRAK1, DISC1/Miro1, TRAK1/Miro1, Kinesin-1/TRAK1 and alpha-tubulin/TRAK1), IP buffer additionally containing 0.1% SDS was used for solubilization of transfected cells and washing of immunocomplexes to obtain high stringency binding conditions. Precleared cell lysates with protein G-Sepharose beads were incubated with anti-Myc mAb, anti-FLAG M2 mAb or anti-RHOT1/Miro1 mAb for 4 h or overnight at 4°C followed by incubation with protein G-Sepharose beads for 2 h to precipitate immunocomplexes.

### Western blot analysis

Protein samples were resolved by SDS–PAGE using NuPAGE 4–12% (Invitrogen). Separated proteins were transferred to polyvinylidene difluoride membranes (GE Healthcare) and then membranes were blocked in T-TBS [50 mm Tris–HCl (pH 7.5), 150 mm NaCl and 0.1% Tween-20] containing 1% skimmed milk for 30 min at room temperature (RT). Incubation with primary antibodies was carried out in the blocking buffer overnight at 4°C. Primary antibodies were used as follows: anti-human DISC1 C-ter pAb (α-DISC1; 1:3000–5000), anti-Myc pAb (1:20 000), anti-FLAG M2 mAb (1:100 000), anti-FLAG pAb (1:10 000), anti-HA mAb (1:5000), anti-RHOT1/Miro1 mAb (1:5000), anti-TRAK1 pAb (1:2000–5000), anti-alpha-tubulin pAb (1:100 000), anti-GAPDH mAb (1:100 000), anti-Kinesin-1 (1:1000) and anti-OSCP (ATP synthase subunit O; 1:1000). Protein bands were detected by incubation with appropriate peroxidase-conjugated secondary antibodies for 45 min at RT and visualized using ECL or ECL-Plus reagent (GE Healthcare). Protein band intensities were evaluated with ImageJ software. All blots shown are representative of multiple independent experiments.

### Immunocytochemistry

COS7 cells were fixed in 3.7% formaldehyde for 10 min at RT followed by fixation/permeabilization with methanol at −20°C for 5 min. To visualize mitochondria, MitoTracker Red CMXRos (Invitrogen) was added to the culture medium at a concentration of 50 nm and incubated for 30 min at 37°C prior to fixation. Fixation of hippocampal neurons (DIV 20) was conducted in 4% formaldehyde (Thermo Scientific) for 10 min at RT then cells were permeabilized with 0.2% Triton X-100 for 5 min. For endogenous DISC1 or Miro1 staining, COS7 and SH-SY5Y cells were fixed in 4% formaldehyde in PHEM buffer (pH 6.9) (60 mm PIPES, 25 mm HEPES, 10 mm EGTA and 2 mm MgCl_2_) for 10 min at 37°C followed by permeabilization with 0.2% Triton X-100 for 10 min. Fixed cells were blocked in phosphate-buffered saline containing 3% bovine serum albumin and incubated with the indicated primary antibodies for 2 h at RT on a platform shaker. Dilution of primary antibodies is as follows: anti-human DISC1 C-ter pAb (α-DISC1; 1:3000-1:4000 for detecting exogenous protein, 1:100 for detecting endogenous protein), anti-FLAG M2 mAb (1:100 000), anti-FLAG pAb (1;10 000), anti-Myc mAb (1:500) and anti-Myc pAb (1:10 000). Alexa Fluor 488 goat anti-rabbit IgG, Alexa Fluor 488 goat anti-mouse IgG, Alexa Fluor 488 chicken anti-goat IgG and Alexa Fluor 647 chicken anti-mouse IgG (1:2000; Invitrogen) were used as secondary antibodies. Confocal images were acquired with a Zeiss LSM 510 microscope (Carl Zeiss) with a Plan-Apochromat 63×/1.4 Oil DIC or a Plan-Neofluar 10×/0.30 objective lens. All images are representative of multiple independent experiments.

### Intensity correlation analysis

COS7 cells expressing Myc-DISC1, Myc-DISC1-Δ47, Myc-DISC1-7RA, Myc-DISC1-37A or Myc-DISC1-37W were double stained with anti-Myc mAb and MitoTracker Red CMXRos. Confocal images were acquired with a Nikon C1SI/TiE microscope equipped with a Plan Apo VC ×100 oil objective. For intensity correlation analysis, WCIF ImageJ bundle was downloaded at http://www.uhnres.utoronto.ca/facilities/wcif/fdownload.html. Images were background subtracted using the BG subtraction from ROI plug-in for ImageJ. The whole cytoplasmic area (except the nucleus) per transfected cell was selected and intensity correlation quotient (ICQ) was calculated using the intensity correlation analysis plug-in for ImageJ to evaluate DISC1/mitochondria staining. The ICQ values, which are distributed between −0.5 and +0.5, are generated as an indication of whether the staining intensities of each channel are associated in a random (ICQ = ∼0), segregated (0 > ICQ ≥ −0.5) or dependent (0 < ICQ ≤ +0.5) fashion (41). Thirty cells from three-independent transfections (10 cells per transfection) were analyzed per sample.

### Isolation of mitochondria

COS7 cells were transfected with Myc-DISC1 or Myc-DISC1-37W plus either FLAG-TRAK1 or empty vector. Mitochondrial fractions were prepared using the Mitochondria Isolation Kit for Cultured Cells (Thermo Scientific) with the reagent-based-method according to the manufacturer's instructions. To obtain extra-purified mitochondrial fractions with reduced contamination from lysosomes and peroxisomes, centrifugation was performed at 3000*g* for the separation from cytosol fractions.

### Antibodies

Primary antibodies used in this study are as follows: anti-Myc mAb (9E10) (Santa Cruz), anti-Myc pAb (Abcam), anti-FLAG M2 mAb (Sigma), anti-FLAG pAb (Sigma), anti-HA mAb (Covance), anti-RHOT1/Miro1 mAb (4H4) (Sigma), anti-alpha-tubulin pAb (Abcam), anti-GAPDH mAb (Millipore), anti-TRAK1 goat pAb (Abcam, used to generate all of the presented endogenous TRAK1 immunoblots), anti-TRAK1 rabbit pAb (Abcam, tested but not used to generate any of the presented endogenous TRAK1 immunoblots), anti-Kinesin-1 pAb (H-50) (Santa Cruz), anti-OSCP (ATP synthase subunit O) and mAb (4C11, Invitrogen). Endogenous DISC1 was detected using anti-human DISC1 C-ter pAb (α-DISC1) (42).

## SUPPLEMENTARY MATERIAL

Supplementary Material is available at *HMG* online.

## FUNDING

This work was supported by grants from the Wellcome Trust (083210/Z/07/Z, 088179/A/09/Z) and the Medical Research Council (G0600214, G0902166), and by RCUK fellowship funding for J.K.M. Funding to pay the Open Access publication charges for this article was provided by the Medical Research Council (G0600214, G0902166).

## Supplementary Material

Supplementary Data
