# Supplementary material for: DISC1 complexes with TRAK1 and Miro1 to modulate anterograde axonal mitochondrial trafficking
Source: Hum Mol Genet. 2013 Oct 2;23(4):906–19. doi: 10.1093/hmg/ddt485 (PMC3900104; doi:10.1093/hmg/ddt485)
Supplement: Supplementary Data [file supp_23_4_906__index.html]

DISC1 complexes with TRAK1 and Miro1 to modulate anterograde axonal mitochondrial trafficking — DISC1 complexes with TRAK1 and Miro1 to modulate anterograde axonal mitochondrial trafficking — DISC1 complexes with TRAK1 and Miro1 to modulate anterograde axonal mitochondrial trafficking — Supplementary Data 

# DISC1 complexes with TRAK1 and Miro1 to modulate anterograde axonal mitochondrial trafficking

## Supplementary Data

Supplementary Data

**Files in this Data Supplement:**

- Supplementary Data - Pdf file
