# Supplementary material for: DISC1 complexes with TRAK1 and Miro1 to modulate anterograde axonal mitochondrial trafficking
Source: Hum Mol Genet. 2013 Oct 2;23(4):906–19. doi: 10.1093/hmg/ddt485 (PMC3900104; doi:10.1093/hmg/ddt485)
Supplement: Supplementary Data [file supp_ddt485_ddt485supp.pdf]

Supplementary Material

DISC1 complexes with TRAK1 and Miro1 to modulate anterograde axonal mitochondrial trafficking

Fumiaki Ogawa[1], Elise L. V. Malavasi[1], Darragh K. Crummie[1], Jennifer E. Eykelenboom[1,2], Dinesh C. Soares[1], Shaun Mackie[1], David J. Porteous[1] and J. Kirsty Millar[1]

[1]University of Edinburgh Centre for Genomics and Experimental Medicine, MRC Institute of Genetics and Molecular Medicine, Crewe Road, Edinburgh EH4 2XU, UK, [2]Now at Centre for Chromosome Biology, School of Natural Sciences, National University of Ireland, Galway, Ireland.

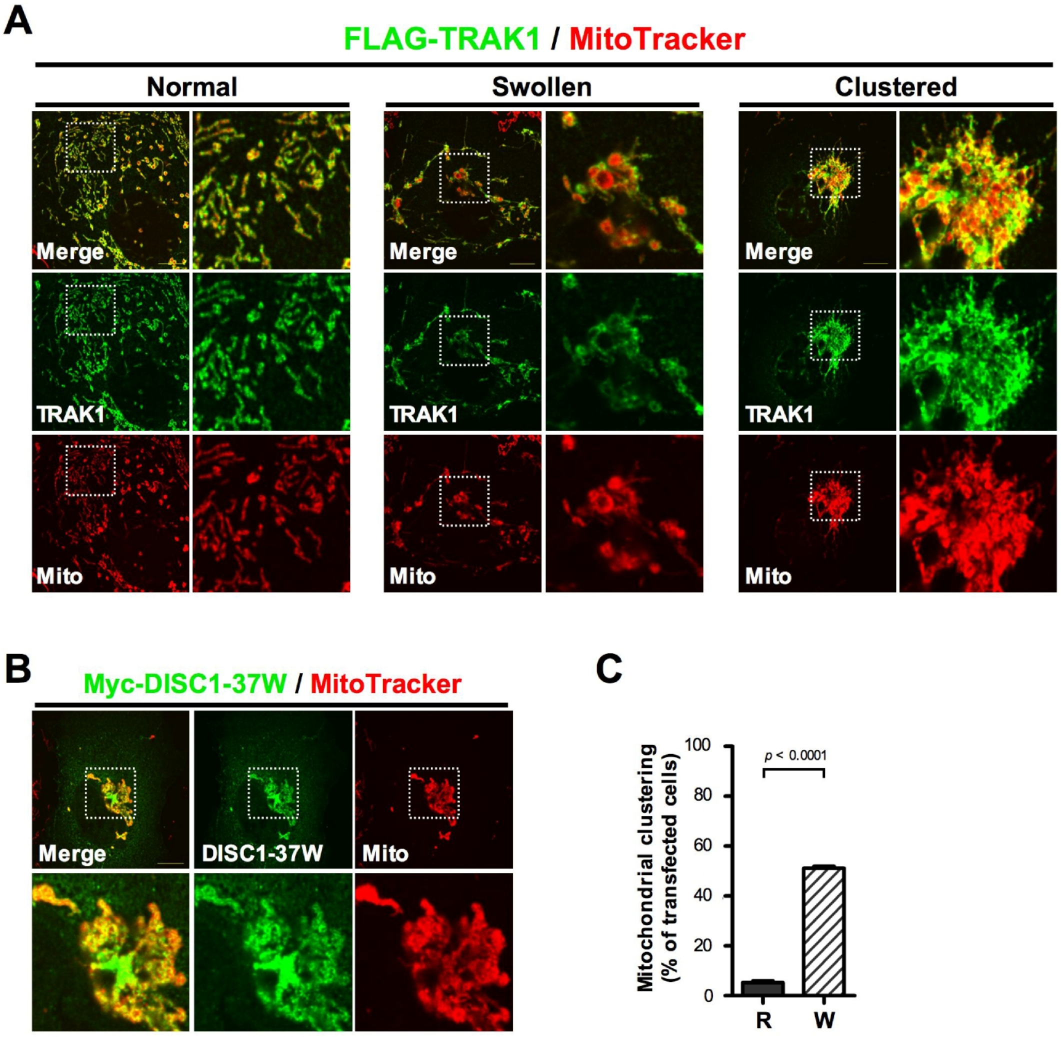

Supplementary Figure S1. TRAK1 and DISC1-37W induce mitochondrial morphological changes. (**A**) COS7 cells were transfected with FLAG-TRAK1 and double-stained with anti-FLAG antibody plus MitoTracker CMXRos at one day post-transfection. Transfected cells typically display one of three mitochondrial morphologies, with mitochondrial clustering apparent in the vast majority of cells. (**B**) COS7 cells were transfected with Myc-DISC1-37W. (**C**) COS7 cells were transfected with Myc-DISC1 or Myc-DISC1-37W and the percentage of transfected cells displaying abnormal mitochondrial clusters was quantified. R: DISC1, W: DISC1-37W. n=3 independent transfections, 300 cells counted per experiment. Error bars represent SEM. Statistical analysis was performed using the (unpaired) two-tailed Student's *t*-test. Scale bars; 10µm.

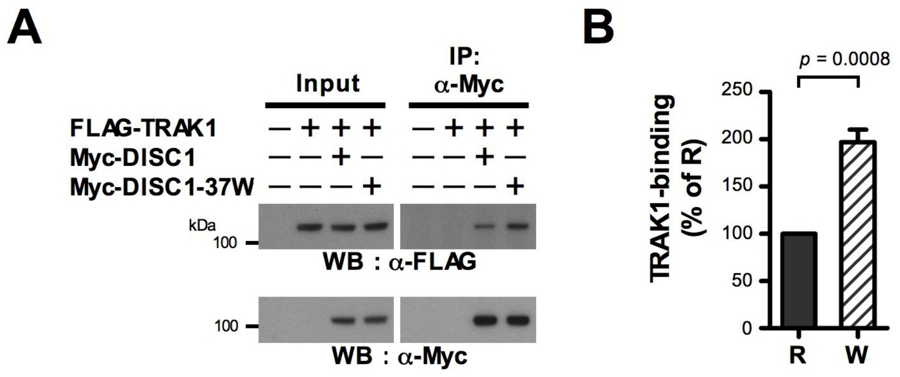

Supplementary Figure S2. Increased binding of the 37W variant to TRAK1. (**A**) Lysates prepared from COS7 cells expressing FLAG-TRAK1 plus Myc-DISC1 or Myc-DISC1-37W, or corresponding empty vectors, were centrifuged at 100,000 *g* to remove insoluble materials and subjected to IP using Myc antibody. (**B**) Quantification of data obtained in A. R: DISC1, W: DISC1-37W. n=6 independent transfections. Error bar represents SEM. Statistical analysis was performed using the two-tailed (paired) Student's *t*-test.

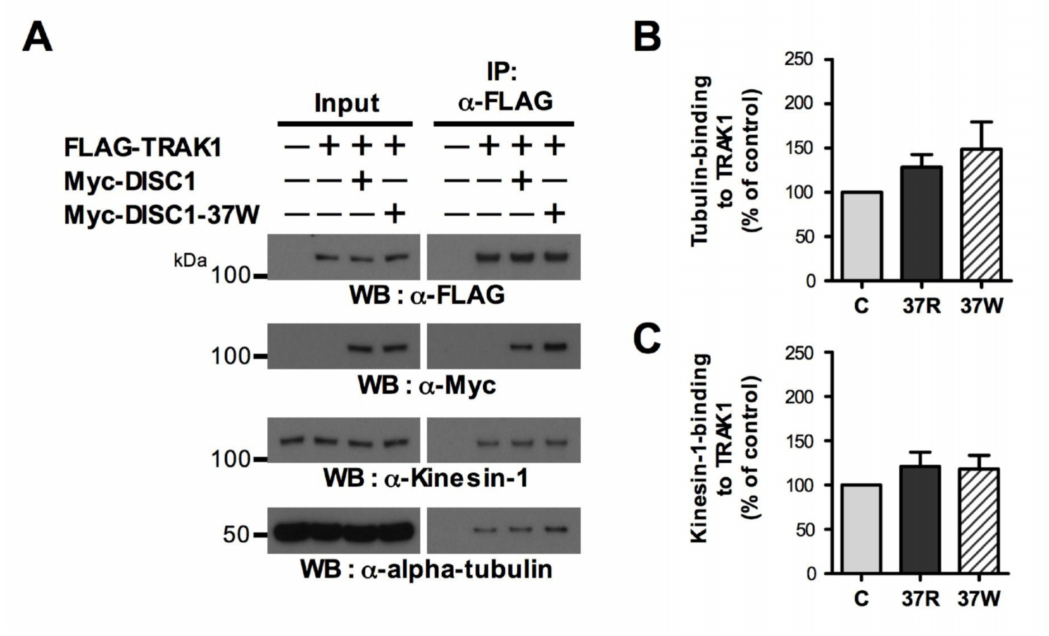

Supplementary Figure S3. Effect of DISC1 upon the association of Kinesin-1 and alpha-tubulin with TRAK1. (**A**) FLAG-TRAK1 was immunoprecipitated, using anti-FLAG antibody, from COS7 cells transfected with FLAG-TRAK1 plus Myc-DISC1 or Myc-DISC1-37W as indicated. Cells that did not receive TRAK1 or DISC1 were instead transfected with corresponding empty vector. (**B** and **C**) Quantification of data shown in A, expressed as ratio alpha-tubulin:FLAG-TRAK1 or Kinesin-1:FLAG-TRAK1, and shown as percentage of that seen in cells transfected with FLAG-TRAK1 plus empty vector. C: empty vector, 37R: DISC1, 37W: DISC1-37W. n=6 independent transfections. Error bars represent SEM.

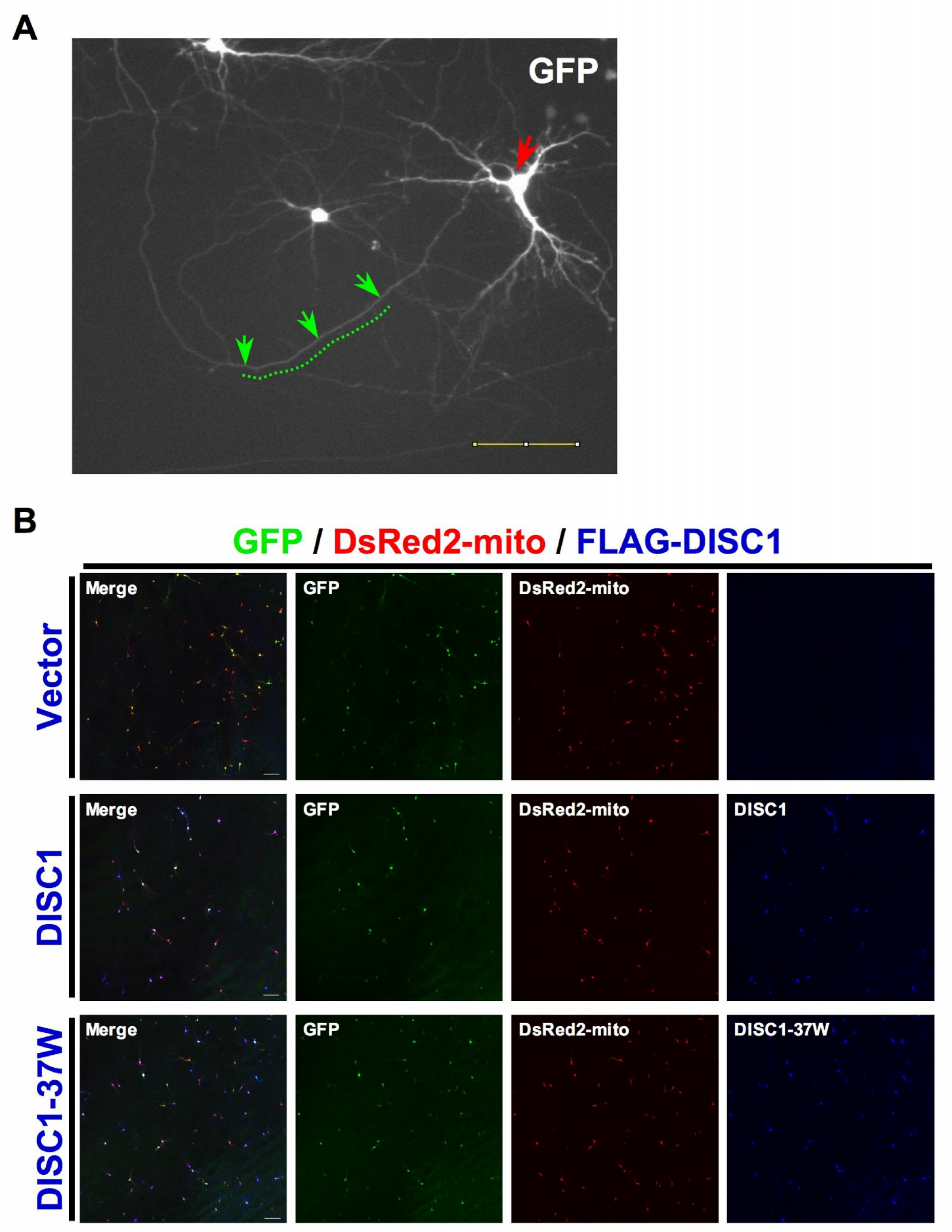

Supplementary Figure S4. Morphological identification of axons and confirmation of neuronal co-transfection rate. (**A**) Representative live cell image of a hippocampal neuron expressing GFP. Axons are easily distinguished from dendrites by their distinctive morphology. Axons have a smaller diameter than dendrites where they originate from the soma (red arrow) (1). Dendrites characteristically terminate 200-300 μm from the soma, while axons (green arrows) are substantially longer. Direction of mitochondrial movement within axonal segments (green dotted line) was determined by examining movement in relation to the position of the soma. (**B**) The majority of GFP^+/$DsRed2\text{-}mito^+$ neurons also express FLAG-DISC1 (DISC1, 97.0%, DISC1-37W, 94.7% over 5 independent experiments). Representative images for retrospective staining of hippocampal neurons transfected with GFP, DsRed2-mito and empty vector, FLAG-DISC1 or FLAG-DISC1-37W. Following live imaging, neurons were fixed and triple-staining was performed using anti-human DISC1 (α-DISC1) antibody. Scale bars; 100 μm.

Reference

1. Kaech, S. and Banker, G. (2006) Culturing hippocampal neurons. *Nat. Prot.*, **1**, 2406-2415.
